# LiverCare - a palliative care intervention for patients with liver cirrhosis: study protocol for a multi-center, pragmatic, non-randomised clinical trial

**DOI:** 10.1186/s12904-026-02182-1

**Published:** 2026-06-16

**Authors:** Malene Barfod O’Connell, Birgitte Gade Jacobsen, Ane Søgaard Teisner, Palle Bager, Marie Louise Hamberg, Janni Mendahl, Kristoffer Marsaa, Mai-Britt Guldin, Nina Kimer, Mette Munk Lauridsen, Lea Ladegaard

**Affiliations:** 1 Gastro Unit, Copenhagen University Hospital, Amager Hvidovre, Denmark; 2 Department of Gastroenterology, University Hospital of Southern Denmark, Esbjerg, Denmark; 3 Department of Gastroenterology and Hepatology, Copenhagen University Hospital, Herlev Gentofte, Denmark; 4 Department of Hepatology and Gastroenterology, Aarhus University Hospital, Aarhus, Denmark; 5 Department of Palliative Care, Copenhagen University Hospital, Herlev Gentofte, Denmark; 6 Steno Diabetes Center Copenhagen, Denmark and Nursing Home Dagmarsminde, Herlev, Graested, Denmark; 7 Center for Grief and Existential Values, Research Unit for General Practice, Aarhus University, Aarhus, Denmark; 8 Department of Clinical Medicine, Faculty of Health Sciences, University of Copenhagen, Copenhagen, Denmark

**Keywords:** Advance care planning, Cirrhosis, Interventions, Implementation, Liver disease, Palliative care.

## Abstract

**Background:**

In the transition from compensated to decompensated cirrhosis, median survival decreases from 12 to two years, and patients face some of the highest rates of unplanned hospital admissions. Despite existing recommendations, palliative care is not routinely offered to this population. The aim of this study is to evaluate the effectiveness and implementation of an interdisciplinary palliative care intervention for patients with cirrhosis and their informal caregivers.

**Methods:**

The LiverCare study is a multi-center, mixed-method, pragmatic, non-randomised clinical study with a hybrid type 1 effectiveness-implementation design. The study is conducted in hepatology departments across four hospitals in three of Denmark’s five regions. It is guided by the British Medical Research Council and Reach, Effectiveness, Adaption, Implementation, Monitor frameworks and includes both a feasibility study and a mixed-method process evaluation.

The LiverCare intervention is grounded in a palliative approach, emphasising open, exploratory palliative care conversations. The intervention is integrated into routine outpatient care and the palliative care conversations are delivered by hepatologists and liver nurses who attended a specially designed course within palliative care. Eligible participants are adults with decompensated liver cirrhosis of any etiology who meet at least one general and one disease-specific palliative care indicator, assessed using the Supportive and Palliative Care Indicators Tool. Informal caregivers are invited to participate in the intervention.

Palliative care conversations are initiated following a formal invitation and continue after four to ten weeks and then every six months up to 36 months or until study withdrawal or death. The primary outcome is healthcare utilization, defined as the number and length of hospital admissions, compared to a historic cohort. Secondary outcomes include outpatient contacts, symptom burden, quality of life, and caregiver burden. The study also includes cost-effectiveness and cost-utility analysis.

**Discussion:**

Insights gained from the study will inform future implementation strategies and contribute to the advancement of palliative care practices in hepatology. The results will further support education, training, and competence development for healthcare professionals caring for patients with advanced liver disease.

**Trial registration:**

The study is registered on clinicaltrials.gov (NCT05431946), approved by The Danish Data Protection Agency and reported to the Committee on Health Research Ethics (20222000-31).

## Introduction

Worldwide, liver cirrhosis ranks as the 11th most common cause of death. In 2019, it accounted for an estimated 1.48 million deaths, reflecting an 8.1% increase since 2017 [1]. The distribution of cirrhosis etiologies and their prevalence varies across countries, largely due to differences in lifestyle factors such as alcohol consumption, obesity, and the risk of viral hepatitis. In Europe, alcohol-related liver disease (ARLD) is currently the leading cause of cirrhosis-related deaths [2].

Liver cirrhosis is the end stage of chronic liver disease of all causes, resulting from sustained inflammation that progresses to fibrosis and, ultimately, liver failure. The progression of liver cirrhosis is divided into a compensated stage, characterized by an absence of specific symptoms and apparent complications, and a decompensated stage featuring one or more severe complications such as ascites, hepatic encephalopathy, infection, renal dysfunction, and variceal bleeding [3]. At this stage, the patient often experiences fatigue, nausea, malnutrition and increasing frailty due to loss of muscle mass. This transition from compensated to decompensated liver cirrhosis significantly worsens health-related quality of life (HRQoL) scores [4, 5], while median survival decreases dramatically from approximately 12 years to just two years [6, 7]. Patients with decompensated liver cirrhosis experience some of the highest rates of unplanned hospital admissions, with 1.5 to 3 liver-related admissions per year [8–12].

The current management of decompensated liver cirrhosis focus on symptom relief, the prevention of further complications, and, if possible, liver transplantation, which remains the only curative option [7–14]. According to the World Health Organization, palliative care should be integrated alongside life-prolonging medical treatment as part of a cohesive approach to care, focusing on enhancing the quality of life for patients and their informal caregivers facing the challenges of serious illnesses. Palliative care aims to prevent and relieve suffering through early identification, assessment, and treatment of physical, psychosocial, and spiritual symptoms and concerns [15].

Given the significant symptom burden, poor prognosis, and high caregiver burden, treatment paths with a focus on early identification of palliative care needs, in combination with life-sustaining treatment and care, should be an integrated part of the treatment and care for patients with decompensated liver cirrhosis and their informal caregivers [16, 17]. Despite existing recommendations, palliative care is not routinely offered to patients with cirrhosis [18, 19], highlighting the need for clinical trials to evaluate the effectiveness and implementation of interdisciplinary palliative care pathways [20].

The aim of this study is to evaluate the effectiveness of an interdisciplinary palliative care intervention for patients with liver cirrhosis and their informal caregivers on key outcomes, including hospital admissions, patient symptom burden, quality of life, and caregiver burden. Furthermore, in an integrated process evaluation we will explore key implementation outcomes, mechanisms of impact, and contextual factors that may influence the interventions success.

## Study design

The LiverCare study is a multi-center mixed-method non-randomised intervention study with a hybrid type 1 effectiveness-implementation design, thus taking a dual focus in assessing clinical effectiveness and implementation [21]. The intervention was developed using a pragmatic design with the aim of utilizing existing infrastructure in the implementation [22]. Furthermore, to ensure a comprehensive understanding of the intervention’s functionality and support potential adaptation or replication, a feasibility study and a mixed-method process evaluation will be conducted.

The study design is guided by the British Medical Research Council (MRC) framework for developing and evaluating complex interventions [23]. Since the MRC guidance does not provide an iterative process or specific research designs, we further utilize the Reach, Effectiveness, Adoption, Implementation, and Maintenance (RE-AIM) Framework, which is efficient for evaluating intervention studies [24]. The RE-AIM framework operates at both the individual level (patients and informal caregivers), and the multiple ecologic level (health care professionals and the centres involved in the intervention).

## Method

### Study setting

The LiverCare study is conducted in four hepatology departments covering three of the five regions of Denmark. The hospitals include Copenhagen University Hospital Amager Hvidovre and Copenhagen University Hospital Herlev Gentofte placed in the Capital Region, University Hospital of Southern Denmark, Esbjerg placed in the Region of Southern Denmark, and Aarhus University Hospital in the Central Denmark Region.

The four hospitals comprise a catchment area of 1.6 million citizens, approximately 27% of the Danish population.

In Denmark, all residents have universal, tax-funded access to healthcare services, including general practitioners and specialized hospital care. The healthcare system is organized into a primary sector (general practitioners and municipal health services) and a secondary sector (hospital-based care) [25].

### Participants and eligibility criteria

#### Inclusion criteria for patients are as follows

- Adult patients with the diagnosis of cirrhosis of any etiology (clinically, radiologically, or histologically confirmed) as the predominant chronic illness.
- At least one general and one disease-specific palliative care indicator assessed by the Supportive and Palliative Care Indicators Tool (SPICT).
- Ability to provide informed consent.
- Ability to read and understand Danish.

SPICT is a needs-assessment clinical tool developed to identify individuals with serious or advanced illness who are at risk of deteriorating health or dying and who may benefit from palliative care [26]. It supports healthcare professionals in recognising potential unmet palliative needs by combining general and disease-specific indicators, although it does not itself determine the exact nature or severity of those needs. In this study, patients were eligible for inclusion if they had at least one general and one disease-specific SPICT indicator.

#### Exclusion criteria include

- Inability to provide informed consent or comply with the study protocol.
- Diagnosis of advanced malignant disease other than hepatocellular carcinoma.
- Current contact with specialised palliative care services or hospice.
- Being listed for liver transplantation.

Eligible patients are invited to participate by a hepatologist or specialised liver nurse, either during hospitalisation or at an outpatient visit. Participation is voluntary, and patients are informed that they may withdraw consent at any time. Patients are encouraged to nominate an informal caregiver to participate in the study. Informal caregivers can include family, close friends, or any other person chosen by the patient. To be eligible, the informal caregiver must be at least 18 years old, be able to speak and understand Danish, and available to participate in at least one study visit. After receiving oral and written information, patients are given at least 24 h to consider participation. If the patient agrees, the first study appointment is scheduled.

## Intervention

### Intervention framework

The intervention is grounded in the modern palliative care paradigm, originating in Cicely Saunders’ concept of “Total pain,” which recognises that suffering has physical, psychological, social, and existential dimensions. These dimensions are interdependent; symptoms are never solely biological but are shaped by personal experience, relationships, and processes of meaning-making. This multidimensional perspective highlights the need to support not only patients but also their informal caregivers, who frequently experience substantial strain when a loved one is seriously ill [27].

Central to the intervention is a person-centred orientation that aligns with the “Platinum Rule” - treat others as they wish to be treated [28]. This requires an understanding of individual values, needs, preferences, and concerns, and a communication approach that actively elicits and responds to these.

To operationalise the palliative approach, the intervention employs the 4-2-4-2 model [29]. Originally developed for chronic lung disease, the model is diagnosis-agnostic and applicable across progressive illness trajectories.

The first four (4–2–4–2) describes four prognostic trajectories as either chronic, early progressive, late progressive, and imminently dying, each associated with distinct goals of care.

The first two (4–2–4–2) denote two forms of knowledge essential in palliative care: objective (e.g., biomarkers, imaging, patient-reported outcomes) and tacit or experiential knowledge that arises through human interaction. Both are necessary for establishing trust, understanding suffering, and tailoring conversations and decisions to each patient and their informal caregivers [30].

The second four (4–2–4–2) revisits the four dimensions of suffering central to the total pain concept: physical, psychological, social, and existential.

The final two (4–2–4–2) refers to the two professional roles required in palliative care: a problem-solving role when suffering is linked to reversible causes, and a companion role when distress arises from irreversible disease processes.

Building on the 4-2-4-2 model, the intervention incorporates an approach to Advance Care Planning (ACP) that operationalises these principles in structured, value-based *palliative care conversations* with patients and informal caregivers. For this study, an approach to ACP that emphasises open, exploratory conversations inviting patients and informal caregivers to share fears, hopes, personal values, and reflections has been adopted. Accordingly, ACP is used not only to discuss treatment ceilings and preferences for end-of-life care, but also to explore broader emotional and existential concerns arising in the context of advanced illness. This expanded orientation reflects the complex and often distressing situations faced by patients and informal caregivers. These conversations are intended to inform the overall plan of care and to ensure that patients and informal caregivers are approached with respect for their values, needs, and dignity.

### Intervention elements

The intervention is delivered alongside standard care and integrated into patients’ regular clinical appointments. In all participating settings, standard care involves routine follow-up of patients with cirrhosis by a team of hepatologists and specialised liver nurses. Following an admission due to decompensated cirrhosis, patients are routinely offered planned follow-up in an outpatient clinic.

The initial palliative care conversation is conducted by a hepatologist in collaboration with a specialised liver nurse. Alternatively, the conversation may be conducted solely by the liver nurse. Subsequent conversations are conducted by either a hepatologist or a liver nurse after 4–10 weeks, and at 6, 12, 18, 24, 30, and 36 months, or until withdrawal from the study or death. Extra visits may be scheduled if clinically indicated or if the patient or informal caregivers request additional support or planning.

#### Invitation

- Patients and their informal caregivers receive an oral invitation to take part in a palliative care conversation about living with cirrhosis and future care preferences.
- Patients and informal caregiver receive a written invitation leaflet designed to support reflection ahead of the conversation.
- Informal caregivers receive written information outlining caregiver participation, acknowledging the burden of caregiving, and providing information on available support services.

#### Palliative care conversations

- Each conversation begins with the healthcare professional setting the stage and clarifying the purpose of the conversation, distinguishing it from routine clinical consultations.
- The conversation addresses individuals’ concerns across the physical, psychological, social, and spiritual domains. It is a collaborative process between patients, informal caregivers, and healthcare professionals to assess and identify problems and symptoms. The patient and caregiver determine the topics discussed and hold most of the speaking time.
- Responses from thorough symptom assessment using the PRO-Pall questionnaire are actively used in the conversation [31]. If the questionnaire has not been completed beforehand, it may be completed during the visit.
- Key topics in the conversations include the daily lives of patients and their informal caregivers, their understanding of the illness, and their hopes and concerns. The conversations provide patients with an opportunity to reflect on what matters most to them, their core values, and their thoughts about future care and treatment.
- Future care is planned, along with actions to be taken based on conversations with patients and informal caregivers, and a timeframe for follow-up is set.

#### Documentation and actions

- The Palliative Care Conversations conclude with a summary of the main points, including agreed-upon care goals and priorities. These are documented in the medical record to inform ongoing care.
- Based on the conversation, the healthcare professional may initiate relevant actions, including but not limited to:
  - Pharmacological treatment adjustments or medication titration.
  - Referral to psychosocial support services (e.g., chaplain, psychologist).
  - Referral to primary care, rehabilitation services, specialized palliative care teams, or hospices.
  - Coordination or communication with informal caregivers, primary care providers, or public services.

## Study overview

**Fig. 1:**
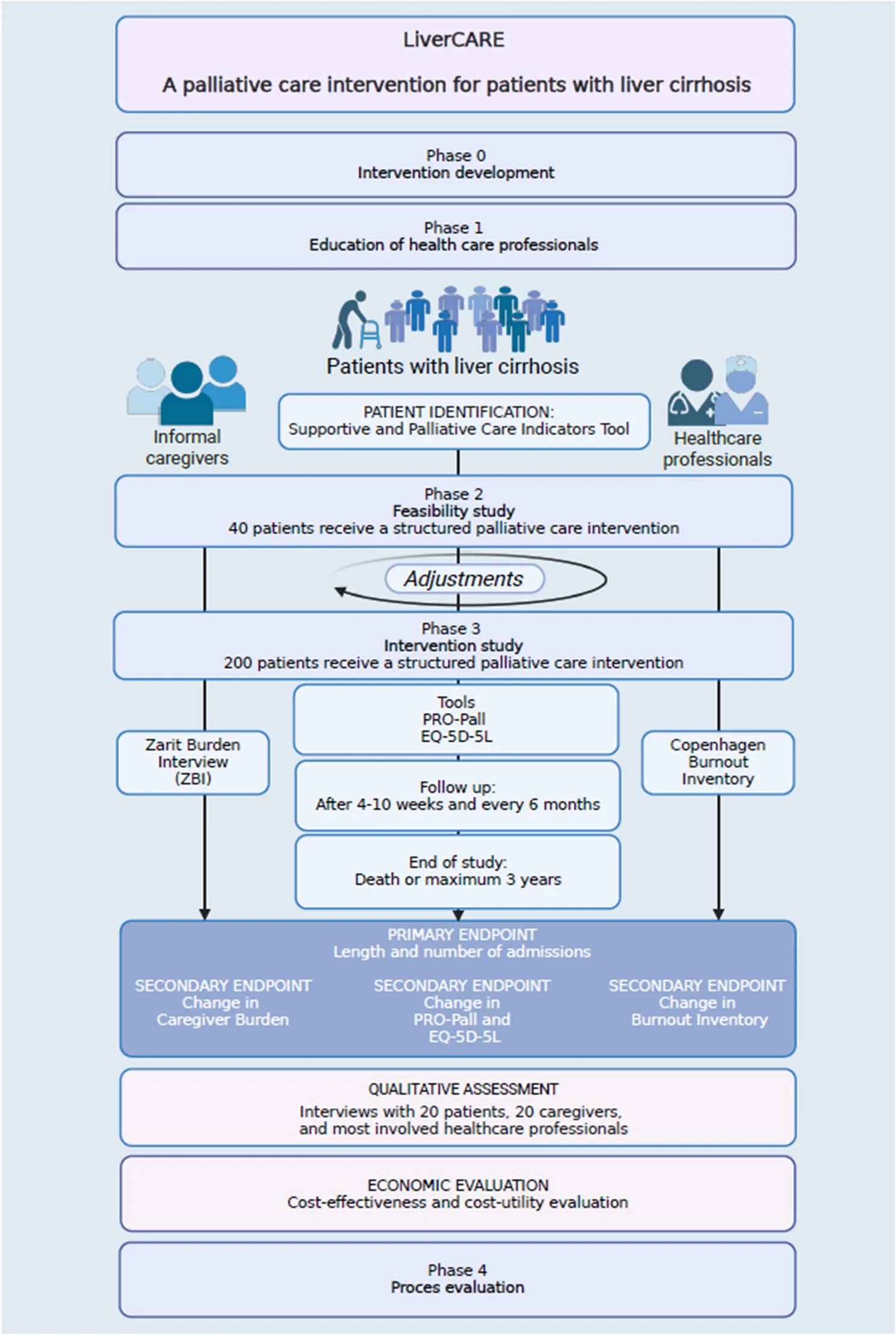
Overview of the LiverCare study

The LiverCare study comprises five Phases, see Fig. 1.

### Phase 0: intervention development

The intervention was developed by the LiverCare Consortium in collaboration with palliative care experts. The LiverCare consortium consists of hepatologists and specialised liver nurses with both clinical and research expertise from the four participating departments, and was established to support a national initiative for palliative care in hepatology.

### Phase 1: education of health care professionals

All healthcare professionals involved in the palliative care intervention attend a specially designed one- or two-day course with a focus on theoretical and practical education and training within palliative care, communication, thorough symptom assessment, PRO-PALL questionnaire [31], ACP, and the 4-2-4-2 model [29, 32]. The education and training were facilitated by palliative care experts and professional actors.

### Phase 2: feasibility study

A mixed-method feasibility study will be conducted to assess whether the palliative care intervention is suitable for further testing in Phase 3. The study will include 40 patients (approx. 10 from each department) and their possible informal caregivers. Findings will be reviewed to adjust and refine the intervention and data collection methods in Phase 3.

### Phase 3: intervention study

A total of 240 patients and possible informal carergivers will participate in the palliative care intervention. The intervention will be initiated following completion of Phase 2, incorporating any adjustments identified during the feasibility phase.

### Phase 4: process evaluation

A mixed method, multistakeholder process evaluation is integrated throughout the study. The process evaluation will be conducted to provide both general and specific recommendations for the continued implementation of the intervention.

## Data collection and outcomes

The data collection and outcome measures are categorised into effectiveness and implementation outcomes, aligned with the research questions.

### Baseline and clinical data

At baseline, sociodemographic data will be collected on participants’ age, gender, highest completed level of education, employment status, housing conditions, family relations, support received from the primary sector, and complicating factors such as substance abuse issues, social network difficulties, financial problems, and housing instability. Additionally, disease-related information will be gathered, including cirrhosis etiology, year of diagnosis, complications, and comorbidities. At each study visit, the patient’s current clinical status will be assessed with particular attention to the severity of cirrhosis-related complications and overall frailty, evaluated using the Clinical Frailty Scale [33]. Liver disease severity and prognosis will be evaluated by calculating the Child-Pugh and Model for End-Stage Liver Disease (MELD) scores [34, 35].

### Effectiveness outcomes

#### Primary outcome

- *Healthcare system burden*, measured as the number and length of hospital admissions from the initial visit to death or study exit. Data on health care utilisation will be prospectively recorded by registering events since the last visit at each study visit and compared to a recent historic cohort of a similar size from the involved centers.

Hospital admissions will be categorised into three mutually exclusive groups:

1. *Liver-related admissions*: inpatient stays of ≥ 24 h due to complications of liver cirrhosis, including variceal bleeding and other bleeding related to portal hypertension, hepatic encephalopathy, spontaneous bacterial peritonitis, hepatorenal syndrome, alcoholic hepatitis, ascites, infection, electrolyte imbalances, increasing frailty, sarcopenia, or poor nutritional status.
2. *Non-liver-related admissions*: inpatient stays of ≥ 24 h for conditions not directly related to cirrhosis.
3. *Short admissions*: hospital stays of < 24 h, excluding admissions in which paracentesis is the sole intervention.

#### Secondary outcomes

**Patients**

- *Outpatient contacts*, grouped into:
  1. *Ascites contact*: outpatient visits or short admissions in which paracentesis is the sole intervention.
  2. *Planned and unplanned clinical visits* in the outpatient clinic.
  3. *Planned and unplanned telephone calls* either initiated by or directed to the patient.
- *Change in symptom burden*, will be assessed using the 24-item PRO-Pall questionnaire [31]. The questionnaire was developed from the 15-item EORTC QLQ-C15-PAL together with nine additional items recommended by the Danish Health Authorities. The questionnaire will be administered at baseline and prior to each planned visit.
- *Change in quality of life*, assessed using the EQ-5D-5 L instrument at baseline and prior to each planned study visit [36].

**Caregivers**

- *Change in caregiver burden*, measured using the 22-item Zarit Burden Interview (ZBI) at baseline and before each planned study visit [37].

**Health care professionals**

- *Change in experience of burnout*, measured using the Copenhagen Burnout Inventory (CBI) before intervention start and one year into Phase 3 [38].

**Health economic outcomes**

- *Cost-effectiveness analysis*, based on the number and length of hospital admissions per patient during the follow-up period.
- *Cost-utility analysis*, assessing the cost per Quality-Adjusted Life Year (QALY) based on the results of EQ-5D-5 L data.

### Implementation outcomes

An overview of the process indicators and methods of measurement for the feasibility study and process evaluation are presented in Table 1.

**Table 1:** Overview of process indicators and methods of measurement and data collection

Domain	Method	Process indicators	Data sources
Reach and uptake	Descriptive quantitative evaluation	Number of patients referred for the intervention Number of patients included of all eligible Referral patterns	Screening data
Effectiveness	Preliminary quantitative evaluation of endpoints in Phase 2 and quantitative evaluation of endpoints in Phase 3 Cost-effectiveness analysis Cost-utility analysis	Number and length of hospital admissions Patient reported outcomes Change in caregiver burden Staff time, training, materials Healthcare utilization QALYs gained	Medical records PRO-Pall EQ-5D-5 L ZBI Danish DRG tariffs EQ-5D-5 L
Adoption		Proportion of sites offering the intervention	Information from study sites
		Differences in implementation between implementation sites	
*Implementation:* Feasibility: *The degree to which the intervention can be carried out in a given setting.*	Mixed-method evaluation Descriptive quantitative evaluation Qualitative evaluation	Resource utilisation Palliative care planning processes No. of invitation leaflets handed out to participants and families No. of palliative conversations per participant No. of informal caregivers participating in palliative conversations. No. of PRO-Pall questionnaires completed for each palliative conversation. No. of medical interventions. No. of patients referred to palliative care teams No. of patients referred to hospices No. of patients referred to municipal offers (exercise, nutrition, alcohol treatment, etc.) Patient experience Caregiver experience Healthcare personnel experience	Self-developed questionnaire with open-ended questions on experiences with palliative care, pre-study training, and the intervention
*Fidelity: The degree to which the intervention was implemented as intended.*			Informal conversations with health care professionals
Acceptability and Appropriateness: *The perception among implementation stakeholders that a given intervention is agreeable*,* palatable*,* or satisfactory.*			Semi-structured interviews informal caregivers and patients
			Medical records
			Semi-structured qualitative interviews
Maintenance	Qualitative and quantitative evaluation	Measures on Reach, effectiveness, after 12 months, two years, three years and five years.	

The feasibility study, Phase 2, explores the acceptability and implementation of the palliative care intervention. Using a mixed-method design and the RE-AIM Framework, the study assesses reach, uptake, fidelity, and preliminary patient and caregiver outcomes across the departments.

Qualitative interviews with patients, informal caregiver, and healthcare professionals will be conducted to assess the intervention’s acceptability and appropriateness. Two subgroups of approx. 20 patients and 20 informal caregivers (five from each study center) will be interviewed to explore their experiences with the intervention. Study nurses and hepatologists involved in delivering the palliative care intervention will be interviewed to assess their perspectives on its implementation and impact as part of Phase 3, the intervention study.

## Sample size and statistical methods

### Sample size

The study aims to include 240 patients and 100 informal caregivers, based on sample size calculations for the most significant outcomes and to accommodate expected attrition.

The Healthcare System Burden will be measured as the number and length of admissions. Patients with cirrhosis spend an average of seven days per year in hospital [39]. Health care utilization data from the intervention group will be compared to a recent historical cirrhosis cohort. For the sample size calculation, we assumed the historical cohort will have an average of seven days in hospital per year and hypothesize that the intervention will cause a reduction of two days/year with an assumed SD of 7 days. Hence, 199 participants are required (STATA BE 18.5). With an estimated attrition rate of 20%, we aim to include 240 patients [40, 41].

Caregiver burden will be measured using the 22-item ZBI (range 0–88). A change of 5 points is considered realistic and clinically relevant. Based on previous studies, this change can be detected with 100 informal caregivers if the standard deviation (SD) of the change is around 14 [35, 36].

### Statistical methods

All data will be entered into a shared REDCap database [42]. Descriptive statistics for numerical data will be presented as means with standard deviations (SD) for normally distributed variables, or as medians with interquartile ranges (IQR) for non-normally distributed variables. Categorical data will be presented as counts and proportions. For between-group comparisons, unpaired two-sample Student’s *t*-tests will be used for normally distributed numerical data, and the Wilcoxon-Mann-Whitney test for non-normally distributed numerical data. Categorical variables will be compared using the chi-squared test. Differences between groups will be reported as means with standard errors (SE) and 95% confidence intervals (CI) for numerical outcomes, and as odds ratios (OR) with 95% CI for dichotomous outcomes. All statistical analyses will be conducted using Stata Statistical Software, version 16 (StataCorp, College Station, Texas). Missing data will be reported and explored. Where appropriate, multiple imputation techniques will be considered. Both intention-to-treat (ITT) and per-protocol analyses will be conducted to assess robustness of findings.

The cost-effectiveness analysis will include cost and effect. The costs will include intervention-related expenses (e.g. staff time, training, materials) while the effect will include the use of healthcare resources using Danish DRG standard tariffs.

The cost-utility analysis will be based on the EQ-5D-5 L-data and the values will be converted to utility scores using Danish population-based value [43]. These scores will be used to estimate QALYs, which will serve as the measure of effectiveness.

The qualitative analysis will follow a systematic approach inspired by content analysis as described by Graneheim and Lundman [44]. An initial overview of each transcript will be obtained through repeated reading. Subsequently, each patient’s experiences and interpretations will be analysed line by line to identify meaning units. Relevant meaning units will be selected, condensed, and coded to reflect the content while preserving context. Codes will be sorted into mutually exclusive categories and subcategories, comparing and describing different experiences regarding knowledge needs across disease etiology and severity [44]. NVivo software (NVivo qualitative data analysis software; QRS International Pty Ltd. Version 12, 2018) is used for the initial sorting of data. The analytical process will alternate between software-based and manual processing to facilitate data management and ensure a comprehensive overview. All categories and meaning units will be re-examined to ensure validity and consistency across the dataset.

## Discussion

The LiverCare study is designed to address the substantial gap in evidence regarding structured, interdisciplinary palliative care for patients with liver cirrhosis. Despite a high symptom burden, high mortality, and marked psychosocial strain, palliative care remains underutilized and poorly integrated in hepatology care pathways [18–20]. Several systemic and cultural factors contribute to this gap, including the unpredictable disease trajectory of cirrhosis, misconceptions equating palliative care with end-of-life care, and limited training among healthcare professionals in addressing the multidimensional needs of this population. By applying a pragmatic, multicenter design, the LiverCare study aims to generate findings with high external validity and real-world relevance, thereby supporting scalable implementation across diverse clinical settings [22].

The intervention explicitly includes informal caregivers, acknowledging the considerable psychological, relational, and practical burden cirrhosis imposes beyond the patient [30–32]. Existing research demonstrates that caregivers experience significant strain and often lack adequate support and information [45]. Enhanced communication and involvement of informal caregivers have been identified as core targets for improving care [45]. By integrating family-centered components and creating structured opportunities for dialogue, the LiverCare study responds to these unmet needs.

A key strength of this study is the hybrid type 1 effectiveness-implementation design, combining evaluation of both clinical and implementation outcomes [21]. Integrating a process evaluation will enable us to distinguish intervention effects from implementation success, explore mechanisms of impact, and assess how contextual factors shape delivery across sites [23]. This is essential in complex intervention research, where positive effects alone do not guarantee sustainable uptake. A failure to recognise the preconditions required to achieve a defined aim may limit generalisability and hinder translation into practice. Implementation science provides the tools to bridge this gap by examining how interventions work in routine systems and identifying what is needed to reach real-world benefit [46].

The inclusion of an initial feasibility study further strengthens the overall study design and reflects the MRC framework’s recommendation to assess feasibility before conducting a full evaluation of complex interventions [23]. Feasibility testing allows us to assess acceptability, fidelity, recruitment pathways, workflow, and data-collection procedures before launching the full trial [47]. These insights will guide refinements of both the intervention and implementation strategy, thereby reducing the risk of attrition, non-adherence, and site-level variation later in the study. Taken together, the feasibility phase and process evaluation increase the likelihood that the intervention will be not only effective but also implementable and scalable.

The multicenter design enhances generalisability but introduces the possibility of variability in prerequisites between sites in terms of culture, staffing, resources, and local workflows. Such heterogeneity may affect fidelity and complicate interpretation of outcome data [23]. The process evaluation is therefore crucial in documenting contextual influences and enabling nuanced understanding of how and why outcomes differ across settings. This knowledge is essential for future scale-up beyond the study sites.

The results of the LiverCare study will be disseminated in peer-reviewed journals and at national and international conferences. Findings will inform clinical practice and support education and competence development for healthcare professionals working with advanced liver disease. Insights will also guide future implementation strategies and contribute to strengthening palliative care provision within hepatology.

## Data Availability

Upon completion of the study, anonymised data will be stored securely in accordance with national data protection regulations. Access to the final dataset may be granted upon reasonable request to the corresponding author, subject to relevant ethical approvals and data protection requirements.

## Abbreviations

ACP: Advance Care Planning
ARLD: Alcohol-related liver disease
CBI: Copenhagen Burnout Inventory
EQ-5D-5L: EuroQol five-dimension five-level questionnaire
HRQoL: Health-related quality of life
MELD: Model for End-Stage Liver Disease
MRC: Medical Research Council
PRO-Pall: Patient-Reported Outcomes in Palliative Care questionnaire
QALY: Quality-Adjusted Life Year
RE-AIM: Reach, Effectiveness, Adoption, Implementation, and Maintenance framework
SPICT: Supportive and Palliative Care Indicators Tool
ZBI: Zarit Burden Interview
